# UAV Mission Planning with SAR Application

**DOI:** 10.3390/s20041080

**Published:** 2020-02-17

**Authors:** Wojciech Stecz, Krzysztof Gromada

**Affiliations:** 1 Faculty of Cybernetics, Military University of Technology, 00-908 Warsaw, Poland; 2 PIT-RADWAR, 04-051 Warsaw, Poland; Krzysztof.Gromada@pitradwar.com

**Keywords:** SAR, UAV path planning, VRPTW, mission planning, MILP

## Abstract

The paper presents the concept of mission planning for a short-range tactical class Unmanned Aerial Vehicle (UAV) that recognizes targets using the sensors it has been equipped with. Tasks carried out by such systems are mainly associated with aerial reconnaissance employing Electro Optical (EO)/Near Infra-Red (NIR) heads, Synthetic Aperture Radar (SAR), and Electronic Intelligence (ELINT) systems. UAVs of this class are most often used in NATO armies to support artillery actions, etc. The key task, carried out during their activities, is to plan a reconnaissance mission in which the flight route will be determined that optimally uses the sensors’ capabilities. The paper describes the scenario of determining the mission plan and, in particular, the UAV flight routes to which the recognition targets are assigned. The problem was decomposed into several subproblems: assigning reconnaissance tasks to UAVs with choosing the reconnaissance sensors and designating an initial UAV flight plan. The last step is planning a detailed flight route taking into account the time constraints imposed on recognition and the characteristics of the reconnaissance sensors. The final step is to generate the real UAV flight trajectory based on its technical parameters. The algorithm for determining exact flight routes for the indicated reconnaissance purposes was also discussed, taking into account the presence of enemy troops and available air corridors. The task scheduling algorithm—Vehicle Route Planning with Time Window (VRPTW)—using time windows is formulated in the form of the Mixed Integer Linear Problem (MILP). The MILP formulation was used to solve the UAV flight route planning task. The algorithm can be used both when planning individual UAV missions and UAV groups cooperating together. The approach presented is a practical way of establishing mission plans implemented in real unmanned systems.

## 1. Introduction

There are many theoretical algorithms for determining UAV flight routes in the literature. Flight planning algorithms for single UAVs, as well as for drone swarms, are described in Viseras et al. (2019) [1]. From the point of view of the algorithms’ detail, they can be divided into general plans of tasks performance and detailed flight schedules. General plans include the allocation of targets for each of the UAVs available during the mission. Detailed flight routes specify the flight route of UAVs. A good review of the literature in this regard is provided in the works of Coutinhoa et al. (2018) [2] and Nunes et al. (2017) [3]. The article discusses the problems of planning a detailed flight schedule, which concerns the calculation of the route for UAVs and the payload (sensors) work plan, which affects the adopted flight route. This task is the most difficult of all tasks belonging to the UAV mission planning group. The model presented in the article in the form of the Mixed Integer Linear Problem (MILP) task allows planners to specify routes for a given number of UAVs, which must recognize a list of targets defined before a mission. However, cooperation between UAVs is not taken into account because each aircraft operates independently, which is a feature for a short-range tactical class UAVs.

It should be noted that detaching the UAV route planning algorithms from the class of systems for which they are made usually causes their uselessness. Depending on the class of unmanned air systems, various restrictions should be taken into account, which significantly change the optimization task related to the determination of flight routes. Similarly, route planning without the payload used for recognition does not guarantee the preparation of a correct flight plan [4,5]. The article will focus on a short-range tactical class system, which in NATO means a system operating at an operating height of up to 5000 m above a sea level and its flight length is usually about a few hours. An example of such a system is Hermes 450, RQ-21 Blackjack or PGZ-19R belonging to the PGZ Group. For UAVs belonging to this class, a set of restrictions related to battlefield operations is defined, which clearly determines the way the mission is planned. The most important limitations determining the way of realizing the reconnaissance mission are: characteristics of reconnaissance sensors (including mainly angles and ranges of observation), times of climbing to a safe cruising altitude, times of reaching the optimum altitude for recognition by a given type of sensor, fuel consumption during altitude change, availability of time corridors for the flight and weather conditions prevailing in the area of recognition (mainly wind speed and cloud cover). When setting routes for UAVs, each of these constraints must be taken into account.

A correct mission plan should meet the mission constraints set for the mission objective. The UAV should complete the mission by traveling from where it is initially located and should follow the plan until it reaches the desired destination. The objective function set for the mission may include, for example, planning the mission along the shortest routes, which leads to reduced fuel consumption. This objective function gives the greatest guarantee that the mission can be completed when the UAVs are not threatened by other dangers. In a situation where movement in dangerous conditions is considered, navigating the shortest routes is not usually a good decision. Minimizing the time to complete the mission is very important, if the mission has time windows imposed on it to recognize specific targets. The most important mission limitation is to minimize the risk of losing UAVs. In military operations, minimizing exposure to or avoiding enemy threats is of utmost importance to the planner and it is critical to the safe and effective performance of the mission by the UAV. In a military environment, UAVs can be detected by the enemy and exposed to being shot down when traveling in hazardous areas.

The article presents some important innovations in mission planning for the short-range tactical class UAV systems. A complete framework of the route planning process for UAVs that are equipped with SAR systems is described. For each phase of the route planning process, methods supporting the development of necessary products are shown in detail. For example, for the route planning phase, a mathematical model was described in the form of a MILP task, the solution of which guarantees optimal routes for a given set of UAVs. In the section presenting results, the algorithms are used for only one UAV, so as not to complicate the considerations, but the model takes into account the operation of multiple UAVs. For the phase of determining the route segments over which the UAV must fly to recognize the target using SAR, a detailed algorithm has been presented, taking into account the parameters of the radar, including synthetic aperture. All calculations were conducted for the commercial SAR military system, which was used in the tests. When planning the route segments, the information obtained from digital maps (Digital Terrain Elevation Data grids) was also taken into account. Without DTED data, it is not possible to plan the correct UAV flight method for target recognition. The algorithm for determining Line of Sight is also presented in the article. All presented algorithms were classified according to the terminology introduced by Coutinhoa et al. (2018) [2].

Based on the nomenclature introduced in Coutinhoa et al. (2018) [2], it can be stated that the article presents the problem of planning a UAV mission for a homogeneous UAV fleet, operating independently or in pairs. A Dubin’s vehicle model has been used. The vehicle’s flight dynamics is neglected. Multiple waypoints must be visited, but each waypoint can be visited only once. Special mission constraints must be considered. (e.g., sensors characteristics, time windows and boundary conditions). The UAVs operate in a 3D space and the obstacles are present. The effects of wind are considered. The problem is not solved in real time. Flight times and velocities are optimization variables.

Section 2 discusses the issues of planning a UAV mission. The most important results in this field were presented. Section 3 presents how to plan short-range tactical UAV missions. The ways to solve the most important planning problems were shown. The VRPTW model used for planning UAV routes is presented in detail, which is adapted to our type of UAV. Section 4 provides some results of planning a UAV mission using experimental data. Section 5 summarizes the results of the work.

## 2. Background

The reconnaissance task for short-range tactical UAVs is hardly present in the literature, although the route planning algorithms, based on VRPTW models, are described in many papers [6,7,8,9,10,11,12,13]. However, the basic issues for these algorithms are ignored, such as the method of constructing the connection network, which takes into account the sensor capabilities, the arrangement of targets to be identified, and threats to UAVs in the terrain. In the case of tactical class systems, the targets defined in tasks are divided into three classes: static (at the time of recognition), dynamic, and Time Sensitive Targets (TST). TST include static or dynamic targets, for which time windows in which they should be recognized are defined. To recognize dynamic objects, one must modify the standard algorithm [14,15]. To correctly plan a route for UAVs, one should also take into account the impact of weather conditions on flight parameters [16].

The basic task of UAV route planning [17] is considered as a Vehicle Route Planning (VRP) class problem, which is a popular combinatorial optimization problem widely used in the areas of distribution and transport [18]. VRP is commonly known as the first time introduced by Dantzig and Ramser (1959) [8].

Nowadays, the UAV routing problem is a new research area that differs from the basic routing of land vehicles. There is a large VRP literature database. A detailed literature query is presented in Toth and Vigo (2014) [12]. In this article, only the literature on UAV routing is reviewed. Due to the scope of the article, various ways of solving the route calculation problem for UAVs, which are an alternative to the exact VRTPW methods based on MILP algorithms, are not described. However, it should be mentioned that there are several methods of finding flight routes for UAVs in the literature, which are based on exact methods and heuristics. MILP-based methods can be modified using a column generation algorithm that is widely described in the literature. The algorithm allows the planner to reduce the time of calculation to obtain a solution by 20%–30% of time of the basic problem. However, due to the complications associated with its formulation, it is not presented in this article. The modified formulation of the problem does not enrich the mission planning method presented in the article.

Heuristic methods are the most common methods related to mission planning described in the literature. They are usually based on genetic algorithms. A significant limitation of these methods is the inability to verify how far from the optimal solution is the feasible solution that is currently found. As in the case of MILP algorithms, there are many articles related to route planning describing how to solve problems using heuristic methods. Another group of algorithms, used for mission planning, are scheduling algorithms, one of which was used to compare with the method proposed in the article based on the MILP class problem. Scheduling algorithms are significantly more effective when tasks are given without an imposed order of execution. However, if one needs to consider time windows for task execution and task priorities, scheduling algorithms become NP-hard problem (Non-Polynomial problem).

The UAV mission planning process involves arranging a flight plan for each UAV available to ensure that the maximum number of targets is recognized, taking into account their importance as described by the priority. The arrangement of the flight plan and the allocation of UAVs for reconnaissance tasks requires target recognition using the sensor type imposed by the commander. In the article, the planning of a reconnaissance mission using a Synthetic Aperture Radar (SAR) is discussed. SAR is a radar used to obtain images of moving objects with high spatial resolution. Such a radar is used to create images of the earth’s surface and the objects located on it. SARs are used for military purposes to recognize objects in the conditions in which optical heads are not useful. The SAR radar also works at a greater distance than the optical head. The details of radar operation are described in Aguasca et al. (2013) [19]. It is noteworthy that SAR radars are a separate group of radars that must be in motion to be able to create an image of the terrain based on the waves emitted and their reflection that returned to the radar. The use of SAR radar requires careful planning of the UAV flight route, due to the way the radar scan is built, the SAR radar must travel straight through a specific UAV flight segment. Planning a UAV route, while taking into account SAR requirements, complicates the mission planning process, as a sensor requires a precisely described pass by the point of interest. A whole route segment must enable data collection: the scanned area cannot be obscured, while being in radar beam, which implies a series of geometrical requirements. The method of determining the flight around the recognized destination is given in Section 3.3.

Unmanned systems equipped with SAR are widely used in civil and military applications for environmental monitoring, mapping, and aerial reconnaissance [20,21]. Compared with traditional SAR air systems, UAV SAR is more flexible in conducting a military reconnaissance mission due to greater safety for UAVs, better reconnaissance parameters, and lower research and development costs. Therefore, the article refers mainly to UAVs equipped with SAR radars when discussing the process of mission planning.

The use of SAR radar has rarely appeared in the literature related to the planning of UAV missions. This is mainly due to the fact that early unmanned systems equipped with SAR were usually mounted on MALE or HALE class drones (the heaviest UAV of above 1 ton of weight) with a high flight height and a high permissible load. Recently, there has been an increased interest in mission planning using SAR [22,23].

When describing a planning process for UAV mission, modern literature addresses the problems of planning UAV flight routes to accomplish their tasks and to avoid threats generated by enemy forces, such as radars or missile systems [6,24]. Many articles in the literature have analyzed UAV route planning in a hostile area of activity and they attempted to assess the risks associated with UAV routes. The risk may be caused by sources of danger or obstacles such as enemy ground-to-air missiles, radar detection zones, no-fly zones, terrain, or airborne vehicles with which UAVs may collide on their routes. Threats can be stationary or mobile, although the majority of articles on this topic concern stationary threats [6,7].

Few papers, focused on mobile threats [25], adopt far-reaching simplifications related to mobile threat modeling. In scientific studies, danger zones are modeled as two- or three-dimensional areas. Xinzeng et al. (2010) [26] have modeled each danger zone as two nested circles. The inner circle represents the no-fly zone and the outer circle represents the extended safety zone. In the article, threats are modeled as three-dimensional rectangles. Thanks to that, the system can verify if the waypoint placed in mission planning phase is above the threat space.

In our research, stationary threat points and stationary targets are used. Threats are modeled as convex polygons or circles. A Voronoi diagram is a very popular graph approach that uses a Delaunay triangulation procedure in order to construct a set of edges around the known threat locations, which can form potential paths from a starting point to a target location [24,27,28]. Triangulation is a very efficient method of path planning in a short distance, but in some cases the results are not acceptable. It is further discussed in Section 3.5. This paper presents an alternative way of modeling the threats based on convex hull calculations.

In the article, it is assumed that there is no obligation to identify all targets that are associated with an increased risk of actions described by the optimization task penalty function. However, this assumption reflects the realities of the battlefield.

## 3. Materials and Methods

### 3.1. Planning a Reconnaissance Mission

In the basic version, the mission planning problem is to find the routes, for each UAV, that minimizes flight time and to ensure recognition of the maximum number of defined targets. This is the basic mission planning task, which is solved at the beginning of planning to verify the feasibility of the mission. This task does not take into account many critical constraints affecting the implementation of the mission, such as the presence of threats in the terrain or the required time windows in which UAVs should reach their destination. These limitations are included in the scheduling algorithm, presented in the paper, which was formulated in the form of a Mixed Integer Linear Problem (MILP) task in Section 3.6.

**Figure 1:**
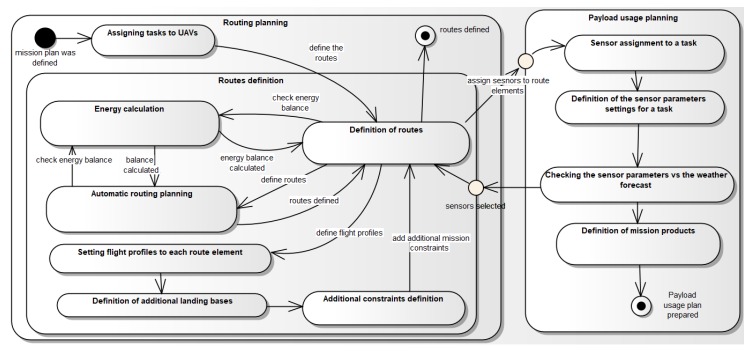
Mission planning scheme.

For the purpose of solving the mission planning problem, the area of operations is modeled in the form of the network S=<V,E>. The vertices V of the network model waypoints, the arcs E of the network model the possibility of UAV flight from the point number *i* to the point number *j*, where i,j∈V, (i,j)∈E. It is assumed that the targets are near selected network arcs, but they are not directly at any waypoint. This is one of the features of tactical class UAVs. Due to their recognition capabilities, they never fly directly above the recognized target. UAV moving along the route, modeled by the arc (i,j) of the network, uses a sensor to recognize the target within the range of this sensor. The method of determining the route sections on the terrain, the UAVs should follow in order to recognize the target optimally, is shown later in the paper. It depends on the type of sensor used to recognize the target. The diagram of the tactical class UAV mission planning process that uses SAR and EO/IR heads is shown in Figure 1.

The route planning task searches for possible routes between all pairs of vertices. The vertices model the points lying near the targets to be recognized. For this purpose, the algorithm for determining the shortest paths between pairs of vertices can be used. Then, the minimum time needed for the transition from the vertex *i* to the vertex *j* is calculated (in the simplest case, only Euclidean distances between individual vertices are taken into account). As a result of determining the shortest paths between pairs of vertices, the square matrix $T_{ij}$ is obtained. In the cells of this matrix, the flight times between destinations are recorded. The indexes i,j∈V, where the set V is the set of vertices of the S network modeling the area of activity between which the UAV moves.

In the planning task, the UAV number selected a priori is assigned to perform recognition. Using terminology from the theory of task scheduling in production processes, each UAV is a machine on which individual operations are performed (recognition of individual targets). The operation is here to identify a specific target.

Flight between waypoints is treated as a changeover (setup) time for the system [29]. The purpose of the problem is to determine the allocation of tasks to the UAV available to recognize all objects. A similar method of determining the flight plan is used by the heuristic tabu search methods [10].

In the literature, it is usually assumed that the targets to be recognized are the point objects (targets are small in relation to the recognition distance), which is a significant simplification and in practice is not acceptable. In the case of surface targets i.e., recognition areas, a UAV flight through such a region should be planned in detail, which in practice generates additional UAV route points. Some guidelines for this type of task are provided in the STANAG 4586 [30] documentation. For the purposes of the work, it can be said that the surface object is modeled in the form of additional vertices, which must all be visited in a given order.

If the constructed network S allows selecting an acceptable schedule, the S network is expanded and a detailed schedule is set.

The process of defining the mission plan is very complex and it consists of many stages presented in Figure 1. This paper omits the problems related to communication planning (data link configuration) and focuses only on a routing process. The requirements of SAR are taken into account too.

### 3.2. Modification of Connection Networks

The target that is recognized in the UAV mission planning task may be the object or area being searched by the UAV. From the point of view of the size of the area, in which tactical class UAVs operate, individual objects such as smaller bridges or buildings are treated as point targets. Larger objects, such as airports or bases, or locations of enemy troops, can be modeled using area targets. For such targets as roads, they are modeled using a set of vertices defining a polygon.

For each of the above types of targets, which will be recognized using a given type of sensor, one must determine the UAV flight segment modeled using the arc (i,j)∈E of the network S. The UAV flight segment in the neighborhood of the recognized target must be located at the optimal distance for reconnaissance. The (i,j) network arc, describing the UAV flight route near the target destination, has got predefined parameters that affect the recognition order. These are the priority of target r∈R recognition $p_{r}$ and the required recognition time (given in the form of the time window $\left[e_{r},d_{r}\right]$, where $e_{r}$ is the earliest date of ISR (Intelligence, Surveillance and Reconnaissance) tasks for the *r*-th target, and $d_{r}$ is the due date of ISR tasks for the *r*-th target.

Each network arc has the defined location ${\left(x,y,z\right)}_{i\in V}$ used for calculating flight times between destinations, the approximate recognition time $t_{r}$ (often referred to as Time on Target for r∈R), the direction of the flight near the target depending on the type of sensor, the preferred flight height, etc. Discussion of all possible parameters used in the recognition process is beyond the scope of this article.

In the case of a linear target, such as a road, the recognition order of the vertices is given (in the form of determining successors and predecessors). Usually, a vertex from which recognition should be started is imposed.

In the case of the vertex modeling the area to be searched, the problem becomes more complicated. There is the whole theory of conducting reconnaissance in the area using UAV, which is reflected in STANAG 4586 [30] and the search patterns themselves are built on the basis of Dubin [31] curves. Each time, it is assumed that a UAV carries out recognition according to the given pattern of action, which significantly affects the time of searching the area [30]. In addition, the time depends on location where a UAV begins the search task and in what location UAV ends reconnaissance. Usually, one or two variants of searching the area are prepared.

In the article, it is assumed that the areas to be recognized are not larger than those that can be scanned by the SAR radar. Thus, no planning of re-flights over the area is needed. Due to the capabilities of modern radars, this assumption does not significantly limit the mission plan (even a small SAR radar can scan a rectangular area with a side length of about 3–6 km).

### 3.3. Scheduling Reconnaissance Using SAR

Radar with synthetic aperture performs a continuous scan during a single passage along one straight route part. During the movement, the radar performs a series of measurements, which are finally processed with appropriate algorithms to generate a single image.

During the radar operation, the relation between the accuracy of the radar positioning on the straight line and the quality of the final result is important. As a result of turbulence, the image loses its sharpness and, in extreme conditions, it may also lose the possibility of the raw data synthesis. Preparation of good scans requires the development of a detailed flight plan near the destination.

It is worth noting that the SAR radar has different requirements for geometrical flight parameters than the EO/IR head. Spatial resolution of image depends on the software settings, not the location of the UAV (which must only be within a certain range of parameters, e.g., for a resolution of 0.15 m, the distance between the UAV and the object must be within a given range over the entire distance, and the flight length on the route segment is usually at least 300 meters.

SAR radars, especially in the case of unmanned aerial vehicles, are permanently attached to the aircraft; therefore, it is necessary to predict the possibility of recording images from one side only.

**Figure 2:**
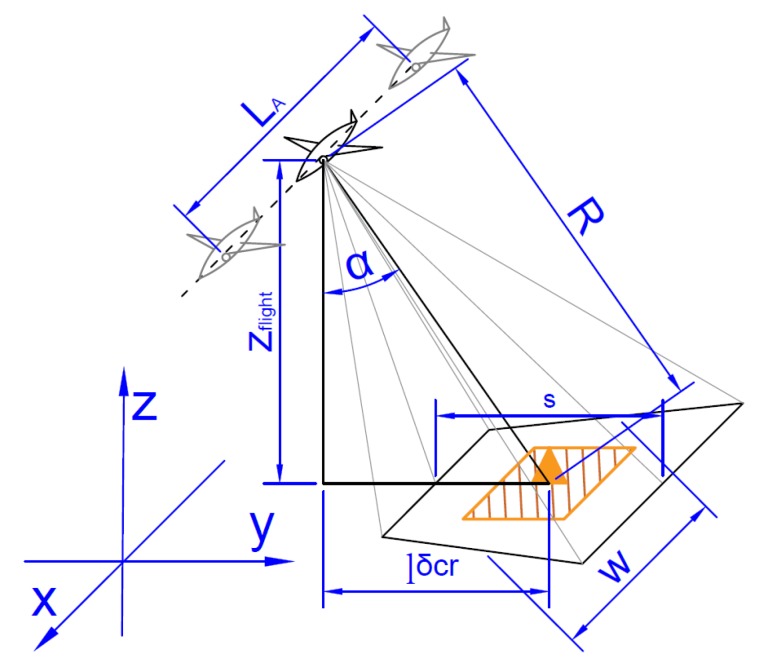
Parameters used for planning SAR radar usage.

Figure 2 shows the basic parameters that should be considered when planning the operation of the radar, which also affects the UAV flight path.

The most important parameters are: α—scanning angle (depends on the construction and installation of the radar), *R*—stand-off range (based on the radar parameters and the required scan resolution), $z_{flight}$—height of UAV above the ground, $l^{\delta_{cr}}$ – projection distance from plane to object, *w*—beam width (derived from α and radar half angle value), *s*—scanning area height, and $L_{A}$—length of the synthetic aperture.

**Figure 3:**
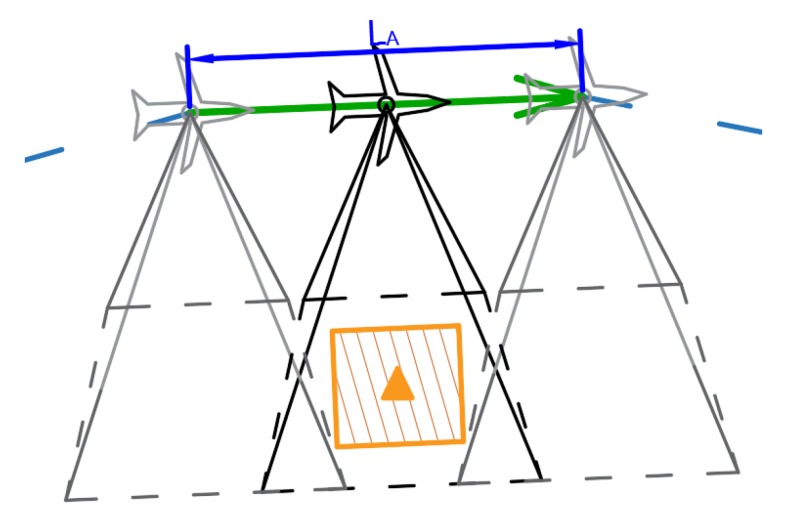
Figure representing geometrical relations between flight segment and scanned POI.

The use of SAR radar to recognize the targets forces the location of the UAV flight route, so that the UAV flight segment has the length of $L_{A}$ and a distance is within the specified distance *R* from the target, and the UAV moves within the specified distance $l^{\delta_{cr}}$ from the recognized destination, where $l_{min}^{\delta_{cr}}\leq$$l^{\delta_{cr}}$$\leq l_{max}^{\delta_{cr}}$ from target. The values $l_{min}^{\delta_{cr}}$ and $l_{max}^{\delta_{cr}}$ depend on the SAR cross range resolution and type of the SAR radar. It is specific for a producer. The process of determining the location of the UAV flight segment is strictly dependent on the sensor used and its integration with the UAV and the sensor construction. An example of route segment planned, taking into account the SAR sensor requirements, with marked beam size, is shown in Figure 3.

The algorithm for calculating the distance $L_{A}$ is shown in Algorithm 1.
**Algorithm 1** Length of the synthetic aperture calculation1:input data: λ (SAR wavelength of transmitted RF centre frequency), κ (SAR radar type)2:set *R* (SAR stand-off range)3:set $\delta_{cr}$ (SAR desired cross range resolution)4:set γ (factor incorporated to account for processing losses)5:$L_{A}=\gamma R\lambda /2\delta_{cr}$6:($l_{min}^{\delta_{cr}}$, $l_{max}^{\delta_{cr}}$) = $f(\delta_{cr},\kappa )$

### 3.4. Determining the Visibility of the Object and Choosing the Direction of Flight

To recognize a target using SAR radar, one must specify $L_{A}$ according to Algorithm 1. As a result of the algorithm, one gets the minimum UAV flight length at a given distance from the target, so that the radar can prepare a SAR scan. A flight segment must have the length between $l_{min}^{\delta_{cr}}$ and $l_{max}^{\delta_{cr}}$ from a target. These lengths are calculated using a predefined SAR scan resolution. The planner’s task is to indicate on the map such a section of the flight route where the SAR radar will prepare the scan. The planner must take into account folding of the area of activity, which may cover the recognized object from the direction of UAV flight.

**Figure 4:**
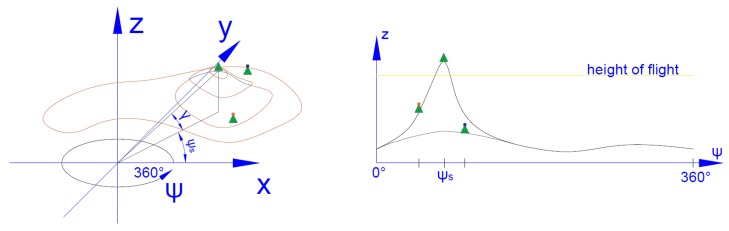
Histogram of terrain elevation in a cylindrical coordinate system. Part of terrain data are presented in the left figure. In the right figure, the histogram for this terrain data are constructed.

In order to determine the possibility of SAR observation of the object from a given direction of flight, it should be checked that the straight line connecting the observed target with the UAV will not be hidden along the entire route by an obstacle between the target and the UAV. For this purpose, a histogram of elevation is created in the reference coordinates system of the observed object for a distance from the UAV to the recognized target (see Algorithm 2). Then, it is possible to prepare data showing the minimum UAV elevation allowing observation of the object from this angle (see Figure 4).

The algorithm for calculating the histogram is shown in Algorithm 2.
**Algorithm 2** Histogram calculation algorithm1:input data: $\delta_{cr}$, $l_{min}^{\delta_{cr}}$,   $l_{max}^{\delta_{cr}}$, $L_{A}$, DTED, r∈R, θ∈(0;1], η=152:**for each** ($dist\in [l_{min}^{\delta_{cr}},l_{max}^{\delta_{cr}}]$) **do**3:      **for each** (Ψ∈[0;360]) **do**4:           select P⊂DTED:P=LoS(r,dist,α)5:           sort P according to the increasing distance from *r*6:           **for each** (p∈P) **do**7:                $\gamma_{p}=atan\left(p,r\right)$;8:                **if** ($\exists l\in P:\gamma_{l}>\gamma_{p},l<p$) **then** $HIDDEN_{p}=true$9:           Ψ=Ψ+θ10:      dist=dist+η;

The algorithm determines a set of points in the neighborhood of the location of the UAV, which will be visible from the target location assuming that the UAV will be flying at a given height. This requires calculation of LoS (Line of Sight), which defines mutual ability for two or more objects to detect each other when there is no obstacle in-between. To check an LoS, one must use the data from the Digital Terrain Elevation Data files. DTED (or Digital Terrain Elevation Data) is a standard of digital datasets which consist of a matrix of terrain elevation values. DTED supports many applications, including Line of Sight analysis, terrain profiling, 3D terrain visualization, and mission planning. In the described experiments, the DTED Level 2 data are used with the accuracy of 30 meters, so η was set to 30.

**Figure 5:**
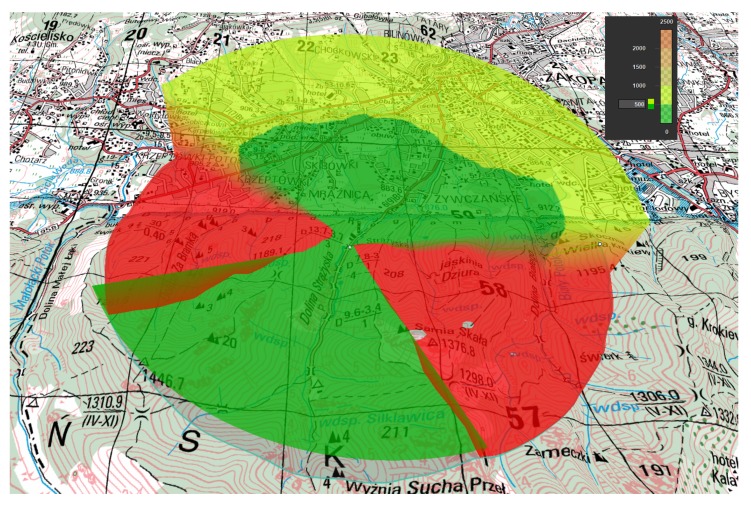
Line of Sight calculation in real terrain. 3D view on the map in 1: 25,000 scale generated by Luciad software.

An example of LoS calculation is shown in Figure 5. The target for recognition is located in the center of the area. The area marked in green indicates places where the target is visible to UAV flying at least 500 m above sea level. The area marked in orange indicates places where the target is visible to UAV flying at least 2000 m above sea level. Flying over the area marked in red will not allow the UAV to recognize the target.

### 3.5. Avoiding Hazardous Areas

There may be zones on the flight path that the UAV should not fly in—so-called prohibited zones. Therefore, the road lengths between sites, modeled with network vertices given in the first stage of planning, must be recalculated. This means that, for each pair of vertices (i,j)∈E, the distance between them should be verified by checking forbidden areas.

**Figure 6:**
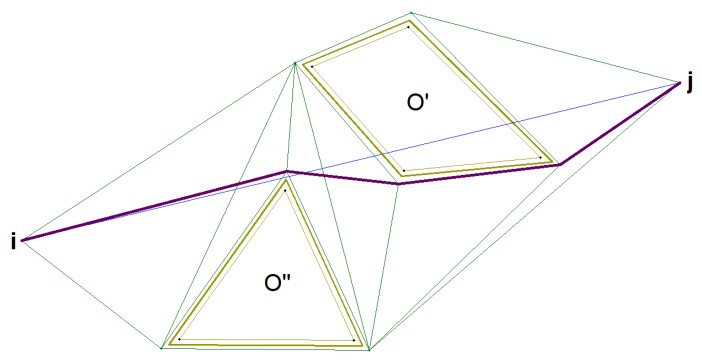
Result of an obstacle avoidance algorithm.

One should then create as short a route as possible, which allows for bypassing the indicated areas (see Algorithm 3—steps 1–2). For the purposes of the article, it can be assumed that all obstacles with the indices o∈O can be approximated by a convex polygon. Additional points are defined around the vertices of these polygons through which the UAVs can pass safely (steps 3–5 of the algorithm). These points are then used to build the graph, specifying the potential paths the object can move on (see Algorithm 4). Figure 6 shows an example of how to calculate a flight route between a pair of the network vertices S. The solution is not based on triangulation and Voronoi diagrams because, in the case of a small number of hazard zones, the determination of roads, based on the Voronoi grid, introduces additional unnecessary UAV long flight segments.

Searching the network, extended by vertices’ modeling dangers (see steps 2–7 of Algorithm 4), allows for finding the shortest path. As shown in Figure 6, a danger zone is defined around each obstacle, which is a polygon surrounding the obstacle. The hazardous area’s offset can be defined by any non-negative value.

The effect of the algorithm is shown in Figure 6, where the bright green line marks the obstacle and the danger area, the waypoints are marked in blue and the flight section (i,j)∈E of the desired route is marked in blue. The graph, showing the possible paths, is marked in green and the shortest path between a pair of the vertices *i* and *j* is marked in purple. As a result of the algorithm for each of the pairs (i,j)∈E, the actual length of the path that UAV must travel to navigate avoiding threats is obtained.
**Algorithm 3** Algorithm for graph construction to avoid obstacles1:**for each**(i,j)∈E:S=<V,E>**do**2:     find o∈O:convexHull(o)∩(i,j)≠∅3:**for each**o∈O**do**4:     extend convexHull(o)5:     generate new waypoints for extended convexHull(o)6:generateTemporaryNet(i,j) for $S^{*}=<V^{*},E^{*}>$ for (i,j)∈E

**Algorithm 4** Temporary net generation and precise path construction
1:generateTemporaryNet(i,j):2:**for each** generated waypoint *w* by o∈O **do**3:    add *w* to the $V^{*}$4:    add (w,l) to the $E^{*}$ where $l\in V^{*}$;5:
**for each** $\left(k,l\right)\in E^{*}$ **do**
6:    **if** ∃o∈O:convexHull(o)∩(k,l)≠∅ **then**7:        $E^{*}=E^{*}-\left(k,l\right)$8:remove subnets of the $S^{*}$ which do not include the vertices $\left(i,j\right)\notin E^{*}$9:find shortest path with Dijkstra algorithm


### 3.6. Algorithm for Determining a UAV Flight Route with Payload Usage

The chapter presents the algorithm for determining UAV flight routes, which is a part of the construction of the mission plan. The algorithm consists of two parts, route planning to verify the possibility of completing the mission and detailed flight route calculation.

The following parameters are used in the optimization task definition.

H—set of indices of UAVs, with *H* elements.
  Each UAV is described by the vector $<z_{h},\tau_{h}>$, h∈H, where $z_{h}$ sets the preferred operational height of UAV and the element $\tau_{h}$ sets a maximum travel time of an unloaded UAV.
V—set of indices of waypoints with *V* elements.
  Waypoint is described by the vector $<{\left(x,y,z\right)}_{i},e_{i},d_{i},p_{i}>,i\in V$, where elements of this vector means:
  (x,y,z)—waypoint coordinates
  *e*—early date (earliest date when an operation or task can start),
  *d*—due date (date when a planned task should be completed),
  *p*—priority.
F—set of airfields for UAV landing with *F* elements,
W=F∪V—set of all waypoints and landing bases indices,
$T_{WxWxH}$—matrix with the dimension WxWxH; element of this matrix represents a time that UAV h∈H needs to fly from the waypoint i∈W to the waypoint j∈W,
$T_{1xV}^{ISR}$—ISR matrix with the dimension 1xV where each waypoint has predefined time needed for intelligence task preparation (i.e., sensor configuration).

**Algorithm 5** Route planning algorithm1:input data: R (set of targets), O (set of danger zones), F (set of airfields)2:**for each**r∈R**do**3:     import vector <${\left(x,y,z\right)}_{r},\left[e_{r},d_{r}\right],p_{r},t_{r}$>4:Find feasible route plan for UAV between the airfields i,j∈F on the net S:5:     Generate the net S where vertices belong to the set W=F∪V using algorithms 1 and 26:     **for each** (i,j)∈W **do**7:          find Euclidean distance between the vertices8:     Find feasible routing for the UAV on the net S with any heuritic algorithm9:     **if** ¬∃ feasible routing **then** there is no solution and exit10:Update distances among vertices in net S using algorithms 3 and 411:Solve VRPTW problem modeled by equations 1–1412:Transform a generated path into a feasible trajectory for the UAV

First, schedule for any UAV can be prepared with the following simple general scenario. Given a set of UAVs and targets, assign each UAV to such a target. It is assumed that each target can have multiple UAVs assigned to it. However, preference is given to high-priority targets. For each UAV assigned, determine an estimated time over the target, taking into account the predefined time window $\left[e_{i},d_{i}\right]$. For each UAV, determine a path via the generated waypoints. UAV must complete the path in the specified time window, while satisfying the given constraints (linked with its maneuvers possibilities and threats). To find the first schedule, for example, the methods presented in Lau et al. (2003) [10] can be used.

After solving the preliminary task, on the basis of a simplified network model, one should solve a complex task that takes into account the terrain elements that affect the change of the UAV flight path. Then, on such a network, one can solve the task of route optimization (point 7 of the algorithm).

The next part of the article presents the VRPTW task in the form of an MILP formulation. It should be noted that any VRPTW algorithm can be used to determine UAV flight routes on the network S presented in the paper, both an accurate algorithm, based on the MILP formulation, and a heuristic one. The decision of whether an algorithm should be used depends on the time to determine the solution. For MILP exact algorithms with a large number of UAVs, things get complicated. In the paper, routes for one or two UAVs are calculated, so it was possible to apply an exact algorithm that worked effectively.

Scheduling problem, based on the general VRPTW problem, is presented in many works, including Li et al. (2018) [21]. In the model presented, the precedence between some waypoints is introduced.

Model variables:

$y_{ijh}$—1 if UAV h∈H travels from waypoint i∈W to waypoint j∈W; 0 otherwise,
$x_{ih}$ — 1 if UAV h∈H travels through waypoint i∈W,
$t_{ih}$—arrival time of UAV h∈H to the waypoint i∈W,
$\mu_{ih}$—technical variable used to eliminate subtours for h∈H path and waypoint i∈W.

Optimization task based on the minimization of the travel time of the UAVs:

(1) $$\rho \underset{i,j\in W}{\sum }\underset{h\in H}{\sum }T_{ijh}y_{ijh}-\underset{i\in V}{\sum }\underset{h\in H}{\sum }p_{i}x_{ih}-\underset{(i,j)\in E}{\sum }\underset{h\in H}{\sum }p_{ij}y_{ijh}$$

subject to:

(2) $$\underset{j\in W}{\sum }y_{ijh}\leq 1\;,\;\forall \left(h\in H,i\in W\right)$$

(3) $$y_{iih}=0\;,\;\forall \left(h\in H,i\in W\right)$$

(4) $$\underset{j\in W}{\sum }y_{0jh}=1\;,\;\forall \left(h\in H\right)$$

(5) $$\underset{i\in W:i\neq j}{\sum }y_{ijh}-\underset{k\in W:k\neq j}{\sum }y_{jkh}=0\;,\;\forall \left(h\in H,j\in W\right)$$

(6) $$\underset{j\in V}{\sum }y_{jbh}=1\;,\;\forall \left(h\in H\right),b\in F$$

(7) $$t_{jh}\geq t_{ih}+T_{ijh}\cdot y_{ijh}-M\left(1-y_{ijh}\right)+x_{ih}\cdot T_{i}^{ISR},\forall \left(h\in H\right),i\in W,j\in W:j\neq i$$

(8) $$x_{ih}\cdot e_{i}\leq t_{ih}\;,\;\forall \left(h\in H,i\in W\right)$$

(9) $$x_{ih}\cdot d_{i}\geq t_{ih}\;,\;\forall \left(h\in H,i\in W\right)$$

(10) $$x_{ih}+x_{jh}-2y_{ijh}\geq 0\;,\;\forall \left(h\in H,i\in W,j\in W:j\neq i\right)$$

(11) $$\underset{j\in W:j\neq i}{\sum }y_{jih}=x_{ih}\;,\;\forall \left(h\in H\right),i\in V$$

(12) $$\underset{i,j\in W:j\neq i}{\sum }T_{ijh}\cdot y_{ijh}+\underset{i\in W}{\sum }x_{ih}\cdot T_{i}^{ISR}\leq \tau_{h}\;,\;\forall \left(h\in H\right)$$

(13) $$\mu_{0h}=0\wedge \mu_{ih}\leq W\;,\;\forall \left(h\in H\right),i\in W$$

(14) $$\mu_{jh}\geq \mu_{ih}+1-W\left(1-y_{ijh}\right)\;,\;\forall \left(h\in H\right),i\in W,j\in W:i\neq j$$

ρ is the optimization coefficient, where ρ∈[0,1]. Constraint (2) ensures that each target is recognized up to once because UAV may visit a vertex only once. Constraint (3) means that the flights within the same vertex are not allowed. Constraint (4) specifies that every UAV must fly on a mission. When the UAV flew into the destination (constraint (5), it must fly out of the destination (to another destination or to the landing base). Each UAV must return to the landing base (index the landing base is b∈W) that is modeled by (6).

Constraints (7)–(9) apply to the time windows of the optimization task. Constraint (7) calculates time of UAV arrival to the waypoint j∈W from the waypoint i∈W. This time must be greater than the time of arrival to the waypoint i∈W plus a travel time between both waypoints and a time needed for reconnaissance task conducted in the waypoint i∈W. $M(1-y_{ijh})$ element of the constraint ensures that fulfillment is met for the waypoints even when $y_{ijh}=0$. Constraint (8) prevents UAV from starting the task before the earliest possible date. Constraint (9) prevents from starting the task after due date.

When UAV travels from i∈W to j∈W, then $y_{ijh}$ equals 1 (constraint 10). When $y_{ijh}$ equals 1, then both waypoints i,j∈W must be visited (constraint 11).

If i∈V represents a route waypoint for h∈H UAV, this UAV must enter this waypoint from any other waypoint from the set W. Maximum travel time of h∈H UAV cannot be longer than $\tau_{h}$, where $\tau_{h}$ depends on the UAV possibility (12).

Constraints (13)–(14) are commonly used to eliminate subtours in VRP—first presented in Miller et al. (1960) [32].

## 4. Results

In the article, UAV mission planning for 10 test cases were tested (see Table 1). One of them concerned mission planning for a UAV, which was to recognize three targets, but there were two areas of danger in the area. The second case concerned the planning of a UAV mission that was to recognize 10 targets in the neighborhood of 10 areas with threats of various types. In both cases, the UAV route could be determined based on the given number of waypoints (usually between 10 and 50). It is usually assumed in military missions that tactical class UAVs have several dozen waypoints defined on their mission, and this number should be less than 100.

**Figure 7:**
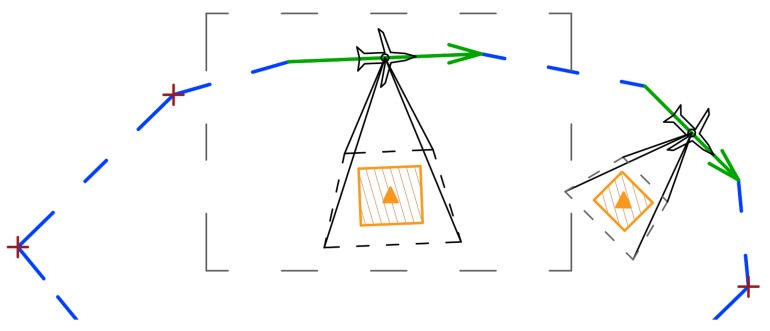
Routing plan for UAV with SAR sensors. Two reconnaissance tasks are presented.

The MILP model, described by Equations (1)–(14), was built based on the CPLEX solver. Figure 7 shows the area of operations where UAV tasks were planned. Targets to be recognized and threat areas for UAVs are marked on the map.

**Figure 8:**
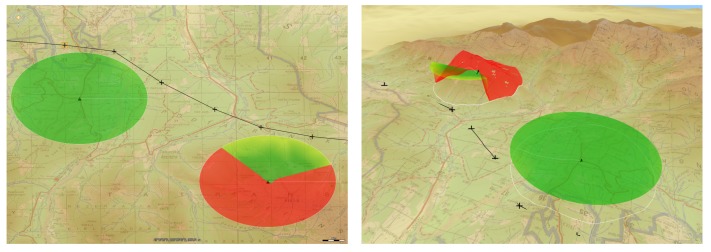
Example of a mission plan presented in 2D and 3D in high mountains.

**Figure 9:**
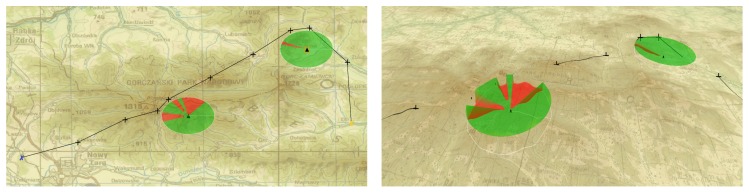
Example of a mission plan presented in 2D and 3D in upland areas.

Figure 8 and Figure 9 present two example plans for missions with SAR usage (presented in Luciad LightSpeed Geographic Information System; maps from http://wcg.wp.mil.pl). Each image was marked with line of sight cone enabling correct data acquisition.

**Figure 10:**
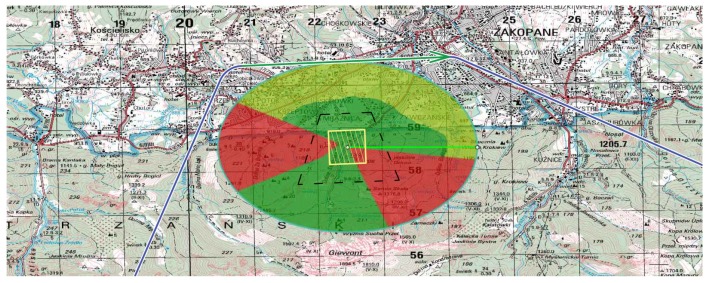
Part of a routing plan for UAV with sensor ISR tasks presented. Accurate flight trajectories determined using the Dubin algorithm.

Figure 10 shows the UAV route determined by the solver and the exact route with the marked flight trajectories taking into account the parameters of the unmanned vehicle used to recognize it.

It should be noted that, if the next waypoints are too close to each other for the UAV, the algorithm may modify the flight route taking into account the UAV turning radius.

Table 1 shows the results of the CPLEX solver, which solved the optimization task (1)–(14) for VRPTW (Vehicle Routing Planning with Time Windows). Calculations were conducted on the personal computer with: i5-2400S-4 * 2,50GHz CPU, GeForce GTX 590 GPU, 4GB DDR3 RAM, 512 GB SSD, and Windows 10 OS.

Cases for different numbers of recognized targets and different numbers of possible waypoints that can be selected as UAV route points are included. It should be noted that the time to solve the route planning task is short enough to be used in combat Ground Control Stations. The calculation time increases nonlinearly if the number of route points increases. On the other hand, increasing the number of pre-defined route segments to be traveled reduces the calculation time, as shown in the table for cases 8–10. This is an important result of the work, since it has been shown that the proposed algorithms are sufficient to calculate the routes for tactical class UAV missions.

The results of the MILP algorithm were compared with the results of the popular and efficient task scheduling algorithm ($1|d_{i}|\sum_{i\in V}\sigma_{i}\cdot \left|L_{i}\right|$) on one machine presented by Brucker and Knust (2005) [29]. In this algorithm, $d_{i}$ means the due time of the task *i* and $\sum_{i\in V}\sigma_{i}\cdot \left|L_{i}\right|$ indicates an optimization function that adds delays to subsequent tasks.

The algorithm can use the values of the time windows for the operations. Assuming identical values of task priorities, the algorithm is more effective than the exact method presented by Equations (1)–(14). However, this method cannot cope with tasks with different priorities. It should also be taken into account that this algorithm is difficult to modify when it is necessary to schedule tasks for more than one UAV.

Comparing the effectiveness of route planning algorithms is difficult because there are no adequate test case databases due to the number of constraints introduced to the described problems. Only the databases for the traveling salesman problem are available, but they are not suitable for testing the described problem.

## 5. Conclusions

The paper presents a method of mission planning for a tactical short-range UAV, which consists of determining the flight schedule taking into account the recognition of targets using the indicated sensors. When building the schedule, there are many additional constraints that were discussed in the article. The most important of them are: the characteristics of the reconnaissance sensor, time windows for recognizing each of the targets, recognition time for any target, availability of air corridors, and the location of enemy forces. However, the most important limitation for setting the schedule is the configuration of the reconnaissance sensor. The paper discusses flight route planning using the SAR radar. Precise determination of the flight plan requires preparation of the modified network S, where the vertices of S model the settings of UAV’s flight parameters or the sensor parameter settings. The network’s arcs model the route elements on which the recognition is carried out.

An important result of the work is to show the usability of VRPTW algorithms for planning the routes of tactical class UAVs. In real reconnaissance tactical systems, actions are not planned for a large scale on a large terrain. Thus, it is possible to prepare an optimization model that will be solved in a short time by the classic MILP solver.

Further work is associated with the introduction of methods for dynamic change of UAV flight plan, which is a particularly difficult task, if you notice that UAV performing reconnaissance task is not usually integrated with other sources of reconnaissance data. This means that extension of the model with elements of dynamic recognition would be associated with expansion of electronic recognition subsystems ELINT on the UAV’s board. Then, functionality of the scheduling system will be strictly dependent on the capabilities of the electronics installed on the UAV. Therefore, the authors maintain the statement, set at the beginning of the article, that the construction of optimization models to solve the route planning task in isolation from the UAV class and its equipment is unacceptable simplification of the problem. 

## Figures and Tables

**Table 1:** VRPTW results for UAV.

Case Number	Number of Targets	Number of Vertices in *S*	MILP	Time of Calculation [s] ($1|d_{i}|\sum_{i\in V}\sigma_{i}\cdot \left|L_{i}\right|$) from [29]
1	1—3	10	2.2	2.0
2	1—3	15	5.4	4.2
3	1—3	20	15.6	10.2
4	3—4	25	10.2	8.1
5	3—4	30	15.3	12.1
6	5—6	35	30.7	20.1
7	5—6	40	45.7	35.2
8	5—6	50	60.7	45.1
9	7—8	50	55.2	47.3
10	9—10	50	50.1	45.6
